# Estimating Underdetection of Foodborne Disease Outbreaks

**DOI:** 10.3201/eid3011.240198

**Published:** 2024-11

**Authors:** Craig W. Hedberg, Melanie J. Firestone, Thuy N. Kim, Alexandra R. Edmundson, Jeff B. Bender

**Affiliations:** University of Minnesota School of Public Health, Minneapolis, Minnesota, USA

**Keywords:** Foodborne outbreaks, bacteria, outbreak size, power law

**To the Editor:** In the February issue, Ford et al. used the power law to estimate underdetection of foodborne disease outbreaks in the United States (*1*). Two of their main conclusions are entirely reasonable: small outbreaks are more likely to go undetected than large outbreaks, and the use of whole-genome sequencing (WGS) has improved the detection of small outbreaks caused by pathogens for which WGS is used. However, their conclusion on the usefulness of the power law itself needs further consideration.

Ford et al. analyzed the size of all foodborne outbreaks reported to the national Foodborne Disease Outbreak Surveillance System during 1998–2019. They defined outbreak size as the number of laboratory-confirmed cases. However, laboratory-confirmed cases are only good estimators for the size of outbreaks detected through pathogen-specific surveillance, such as for *Salmonella*, where outbreak detection follows the accumulation of confirmed cases. For outbreaks associated with events or establishments, identification might rely on reports from consumers, many of whom do not seek healthcare; thus, stool specimens might only be collected from a few cases to confirm the etiology. Consumer complaints are the primary means for identifying foodborne outbreaks caused by norovirus. The Council to Improve Foodborne Outbreak Response recommends collecting clinical specimens from >5 members from the ill group in such settings (*2*). Thus, the number of confirmed cases in an outbreak is dependent on how the outbreak is detected. Outbreaks detected by complaint generally have few confirmed cases, even though they can involve large numbers of illnesses.

To provide a fair evaluation for the usefulness of the power law, it may be better to restrict analyses to outbreaks with common detection pathways. For outbreaks detected by pathogen-specific surveillance, counting confirmed cases seems appropriate. For outbreaks detected through consumer complaints, analyses should include all outbreak-associated illnesses.
